# Low Dose of Sucralose Alter Gut Microbiome in Mice

**DOI:** 10.3389/fnut.2022.848392

**Published:** 2022-02-25

**Authors:** Zibin Zheng, Yingping Xiao, Lingyan Ma, Wentao Lyu, Hao Peng, Xiaorong Wang, Ying Ren, Jinjun Li

**Affiliations:** ^1^Hubei Key Laboratory of Animal Nutrition and Feed Science, Wuhan Polytechnic University, Wuhan, China; ^2^State Key Laboratory for Managing Biotic and Chemical Threats to the Quality and Safety of Agro-Products, Institute of Agro-Product Safety and Nutrition, Zhejiang Academy of Agricultural Sciences, Hangzhou, China; ^3^Institute of Food Sciences, Zhejiang Academy of Agricultural Sciences, Hangzhou, China

**Keywords:** sucralose, low dose, gut microbiome, mice, intestinal barrier

## Abstract

Sucralose is a non-nutritive artificial sweetener (NNS) used in foods or beverages to control blood glucose levels and body weight gain. The consumption of NNS has increased in recent years over the world, and many researches have indicated long-term sucralose administration altered the gut microbiome composition of mice. These studies all focus on the US Food and Drug Administration (FDA) defined acceptable daily intake (ADI), approximately 5 mg/kg BW/day for human. In our study, mice were given with T1-4 (0.0003, 0.003, 0.03, and 0.3 mg/mL) of sucralose, respectively, Control group mice were given normal water. In particular, 0.3 mg/mL of sucralose was equal to the ADI (5 mg/kg BW/day). After 16 weeks, all mice were weighted and sacrificed, the liver of each mouse was isolated and weighed, segments of jejunum, ileum and colon were collected for H&E-stained. The contents of jejunum, ileum, cecum and colon were collected for 16S rRNA gene sequencing. The results showed sucralose administration affects the intestinal barrier function evidenced by distinct lymphocyte aggregation in ileum and colon while not change the mice body weight. The 16S rRNA gene sequencing of the mice gut microbiome suggested sucralose administration significantly changed the composition of gut microbiota, especially in T1 and T4 group. For example, a reduction of probiotics abundance (*Lachnoclostridium* and *Lachnospiraceae*) was found in cecum of T4 group mice compared with Control group. On the other hand, *Allobaculum*, which was reported positively correlated with diabetes, was increased in the T1 and T4 group. In addition, the potential pathogens, including *Tenacibaculum, Ruegeria, Staphylococcus* were also increased in jejunum, ileum and colon by sucralose administration in T1 and T4 group. These new findings indicate that low dose of sucralose (T1) alter gut microbiome in mice, and these adverse health effects are equal to ADI level (T4). Overall, our study provides guidance and suggestions for the use of sucralose in foods and beverages.

## Introduction

Global consumption of sugar-free foods is increasing. Non-nutritional sweeteners (NNS) added to beverages and foods are defined as sweetener with higher sweetness and lower calorie content than caloric or nutritional sweeteners (such as sucrose or corn syrup) (1). Sucralose also named trichlorogalactosucrose and TGS, is a NNS, zero-calorie artificial sweetener (2). It is a substitute for chlorinated sugar, and its sweetness is 600 times than sucrose, because of its low production cost, high thermal stability and solubility, sucralose has become an important sugar substitute in foods and beverage (3, 4). US Food and Drug Administration (FDA) defined acceptable daily intake (ADI) approximately 5 mg/kg BW/day for human (5, 6). The adverse health effects of sucralose have been highly argued over the years. For example, a large number of early studies have shown that most of the ingested sucralose will not be absorbed and metabolized by the body, and it will not change with gut peristalsis (7, 8). However, researches have confirmed that sucralose can change the composition of gut microbiome, inhibiting intestinal development, and aggravating HFD-induced hepatic steatosis in adulthood (5, 9).

Gut microbiome refers to the complex community of microorganisms living in the digestive tract of human and animals, its number is about 10 times than our body cells (10). The balance between host and gut microbiome is essential to maintain a healthy gut barrier and optimal immune homeostasis, which helps to prevent the occurrence of diseases (11, 12). Gut microbiome contribute to the metabolic health of the human host, when aberrant, it will cause the pathogenesis of various common metabolic disorders including obesity, type 2 diabetes, non-alcoholic liver disease, cardio-metabolic diseases and malnutrition (13). Related research used fecal samples from Sprague Dawley rats that received artificial sweetener sucralose (1.1%) for 12 weeks, the results show that sucralose administration reduced the total number of anaerobic bacteria, aerobic bacteria, bifidobacteria, Lactobacillus, Bacteroides and Clostridium (14). Uebanso research showed that the abundance of Clostridium flora in the high-dose sucralose group decreased significantly, and the concentrations of butyric acid and bile acid increased in a dose-dependent manner with the intake of sucralose (15). So, sucralose administration significantly altered mice gut microbiome, and reduced the abundance of beneficial bacteria.

Although many studies have deeply explored the impact of sucralose on gut microbiome, most studies were close to the concentration of ADI (5 mg/kg BW/day) (16, 17). In this study, we found low concentration sucralose also significantly altered gut microbiome by setting four concentration gradients, and it might involve in the development of diabetes. It provides a research basis for the adverse effect mechanism of sucralose on human health, and provides guidance and theoretical support for the practical application of artificial sweeteners.

## Materials and Methods

### Animals and Sampling

Forty specific pathogen-free (SPF) C57BL/6J male mice weaned at the age of 28 days were purchased from SLAC Laboratory Animal Co., Ltd (Shanghai, China). The mice were raised in cages at 25 ± 2°C for 12 h light/dark cycles with free access to water and mouse chow. After acclimatization for 1 week, the mice were weighed and randomly divided into 5 groups and treated for 16 weeks as follows: Control group mice were given distilled water (C, *n* = 8), Trichlorogalactosucrose (TGS) 1–4 groups mice were given a sucralose solution of 0.0003 g/mL (T1, *n* = 8), 0.003 mg/mL (T2, *n* = 8), 0.03 mg/mL (T3, *n* = 8), 0.3 mg/mL (T4, *n* = 8) per day. FDA defined ADI for sucralose in humans were 5 mg per kg (body weight) (18), 0.3 mg/ml is equal to a mouse with an average body weight of 0.02 kg, according to the following calculation:


$$\begin{array}{l} \frac{\text{ADI }5\text{mg}/\text{kg}/\text{day }\times \text{ average mouse weight }0.02\text{Kg}}{\text{Average daily liquid intake }3\text{ml}}\\ \;\approx 0.3\;mg/mL \end{array}$$

At the end of 16-week study, all mice were weighed individually and euthanized. The liver of each mouse was isolated and weighed. Segments of jejunum, ileum and colon were collected and fixed in 4% paraformaldehyde for H&E-stained. The contents of jejunum, ileum, cecum and colon were collected and stored at −20°C until DNA isolation and 16S rRNA gene sequencing. More detailed bioinformatics methods can be found in a previous study (19).

### Histological Staining

The jejunum, ileum, and colon tissue segments (1 cm) were collected for histological staining from 3 mice per group, the tissues were rinsed with PBS, immediately fixed in 4% paraformaldehyde, and then cut into sections (4–5 mm), the H&E staining were used to stain the tissue sections according the methods described by previous study (20).

Histopathological scores were calculated according to the methods described by Ma (21): Epithelial surface loss, crypt destruction, and immune cell infiltration (0: no change, 1: localized and mild, 2: localized and moderate, 3: localized and severe, 4: extensive and moderate, 5: extensive and severe).

### DNA Extraction and PCR Amplification

Microbial genomic DNA was extrtacted from each intestinal content according to the manufacturer's instructions (QIAamp DNA Stool Mini Kit QIAGEN, CA). The V4-V5 region of the bacteria 16S ribosomal RNA gene was amplified by PCR (95°C for 2 min, followed by 25 cycles at 95°C for 30 s, 55°C for 30 s, and 72°C for 30 s and a final extension at 72°C for 5 min) using primers 515 F 5′-barcode- GTGCCAGCMGCCGCGG)-3′ and 907 R 5′-CCGTCAATTCMTTTRAGTTT-3′, where the barcode is an eight-base sequence unique to each sample. The PCR reactions were performed in triplicate using 20 μL mixture which contained 4 μL of 5 × FastPfu Buffer, 2 μL of 2.5 mM dNTPs, 0.8 μL of forward primer (5 μM), 0.8 μL of reverse primer (5 μM), 0.4 μL of FastPfu Polymerase, 0.2 μL of BSA and 10 ng of template DNA, then add ddH_2_O to 20 μL. Amplicons were extracted from 2% agarose gels and purified using the AxyPrep DNA Gel Extraction Kit (Axygen Biosciences, Union City, CA, U.S.) according to the manufacturer's instructions and quantified using QuantiFluor™-ST (Promega, U.S.).

### Library Construction and Sequencing

Purified PCR products were quantified by Qubit^®^3.0 (Life Invitrogen) and every 24 amplicons whose barcodes were different were mixed equally. The pooled DNA product was used to construct Illumina Pair-End library following Illumina's genomic DNA library preparation procedure. Then the amplicon library was paired-end sequenced (2 × 250) on an Illumina Novaseq platform [Mingke Biotechnology (Hangzhou) Co., Ltd] according to the standard protocols. The original image data files obtained by high-throughput sequencing were converted into Sequenced Reads by Base Calling analysis, the results were stored in FASTQ (referred to as fq) format file, which contains sequence information of reads and their corresponding sequencing quality information.

### Statistical Analysis

All statistical analyses were performed by SPSS 23.0 (IBM, New York, NY, United States) using One way ANOVA (22). Data are presented as the mean ± SEM. Results were considered significant when *P* < 0.05.

## Results

### Sucralose Administration Did Not Change the Phenotype of Mice

**Figure 1:**
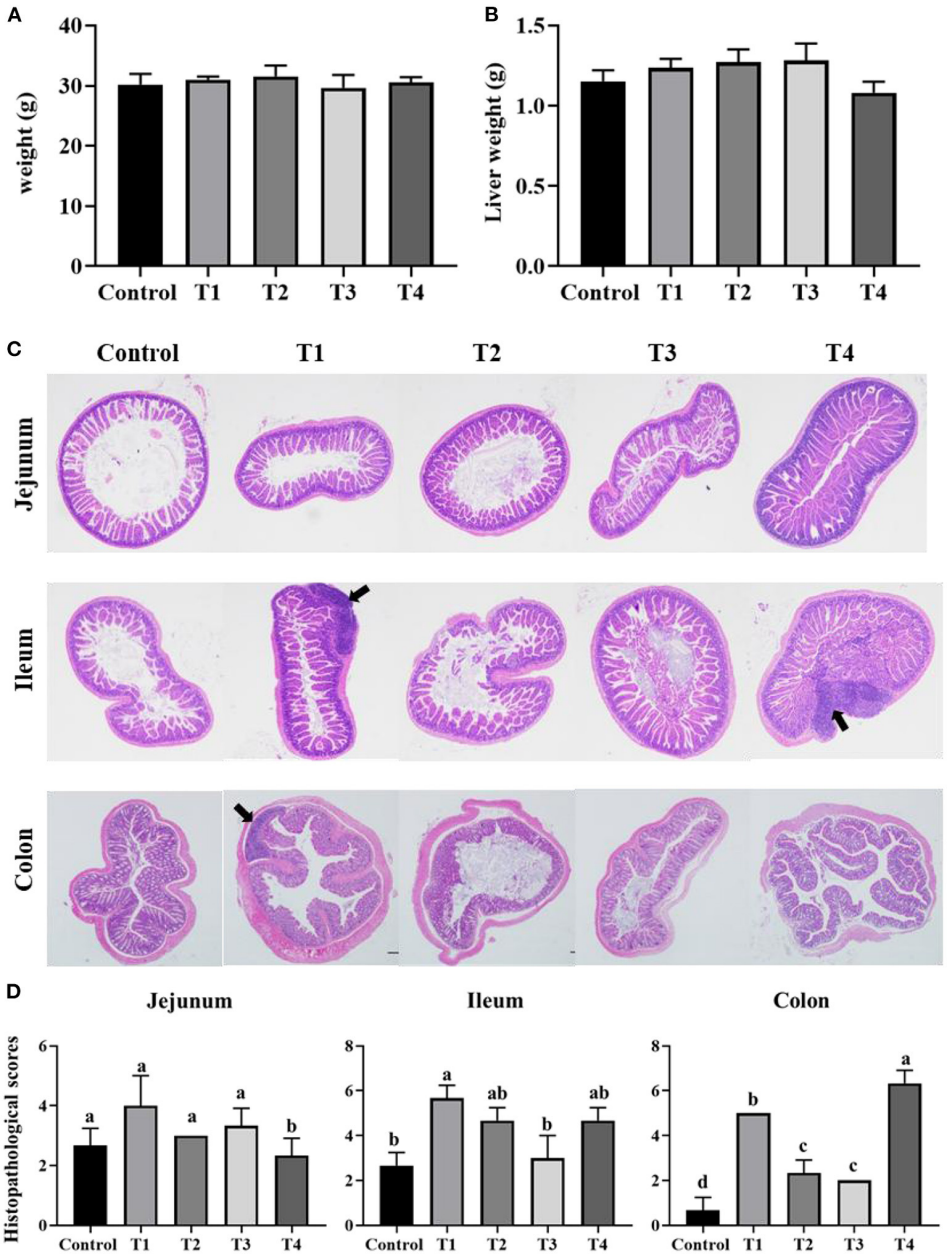
Bar chart showing the mice body weight **(A)** and liver weight **(B)**. Representative H&E-stained sections from jejunum, ileum, cecum and colon **(C)**, the arrowhead points in the direction where lymphocyte aggregation. Histopathological scores of the H&E staining **(D)**. Data was expressed as mean ± SEM (*n* = 8) and analyzed by one-way ANOVA analysis. The different superscript letters on the histogram represent a significant difference **(D)** (*P* < 0.01).

In order to confirm the effect of zero-calorie sucralose on body nutritional absorption, Mice was given with T1-4 (0.0003, 0.003, 0.03, and 0.3 mg/mL) of sucralose in drinking water, respectively. The results showed mice body weight and liver weight were not significant differences between Control group and T1–T4 groups (Figures 1A,B). To observe the effect of sucralose on intestines of mice. We stained mice intestinal tissue segments of each group. Compared with the Control group, the intestinal barrier and goblet cells of T1-4 groups were significantly damaged, and there was distinct lymphocyte aggregation in ileum and colon of T1 group and ileum of T4 group (Figure 1C). According to the scores of H&E staining, T1 and T4 group presented with severe acute colitis, crypt destruction, disappearance of superficial epithelial cells and goblet cells, and the increased infiltration of inflammatory cell (Figure 1D).

### Sucralose Administration Altered Mice Gut Microbiome

**Figure 2:**
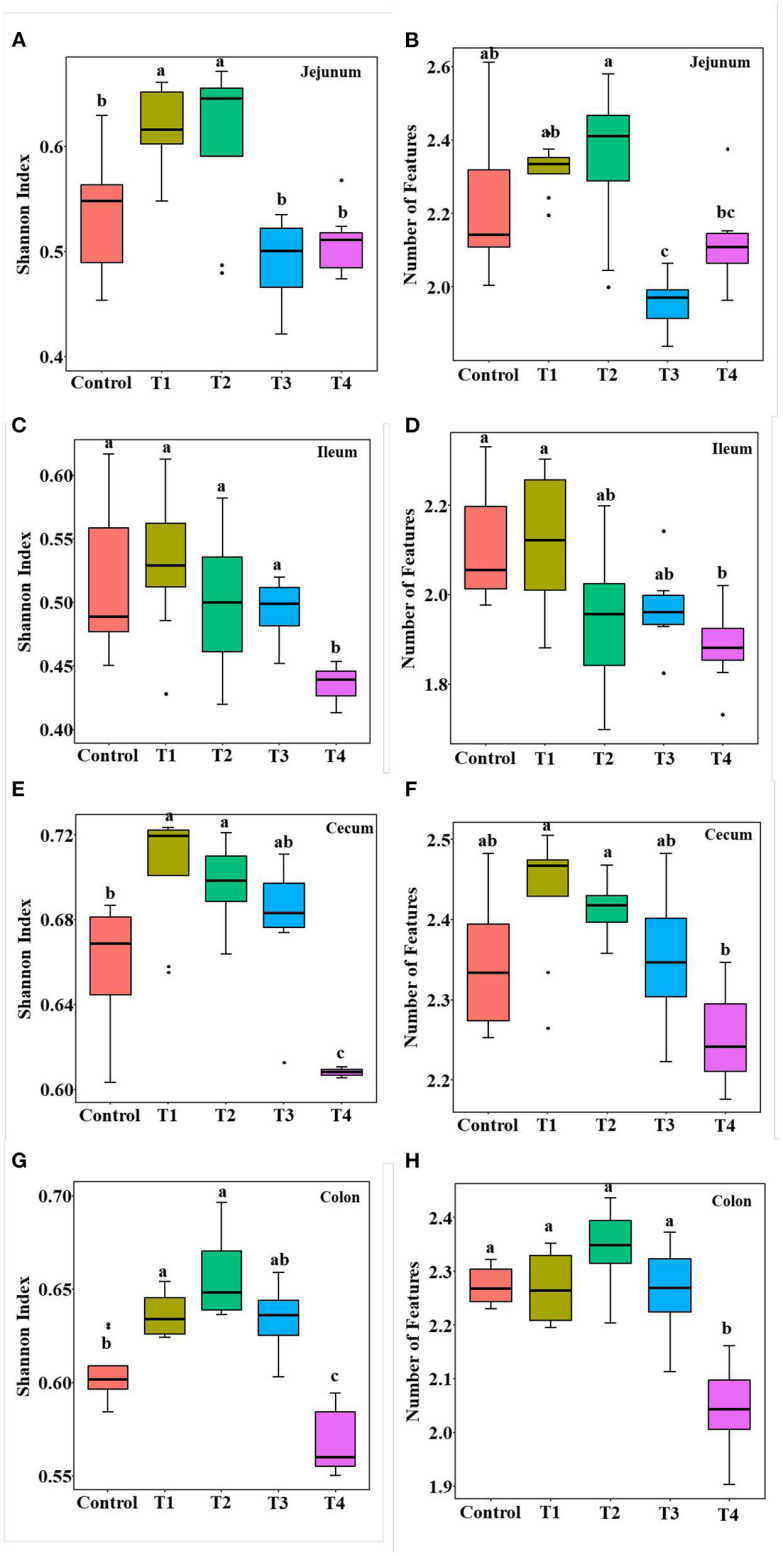
Alpha diversity including Shannon index **(A,C,E,G)** and the number of features **(B,D,F,H)** in control group, Trichlorogalactosucrose (TGS) 1–4 (T1, T2, T3, T4) groups in jejunum **(A,B)**, ileum **(C,D)**, cecum **(E,F)**, colon **(G,H)**. Data was processed through log10, and expressed as mean ± SEM (*n* = 8) and analyzed by one-way ANOVA analysis. The different superscript letters on the boxplot represent a significant difference (*P* < 0.01).

**Figure 3:**
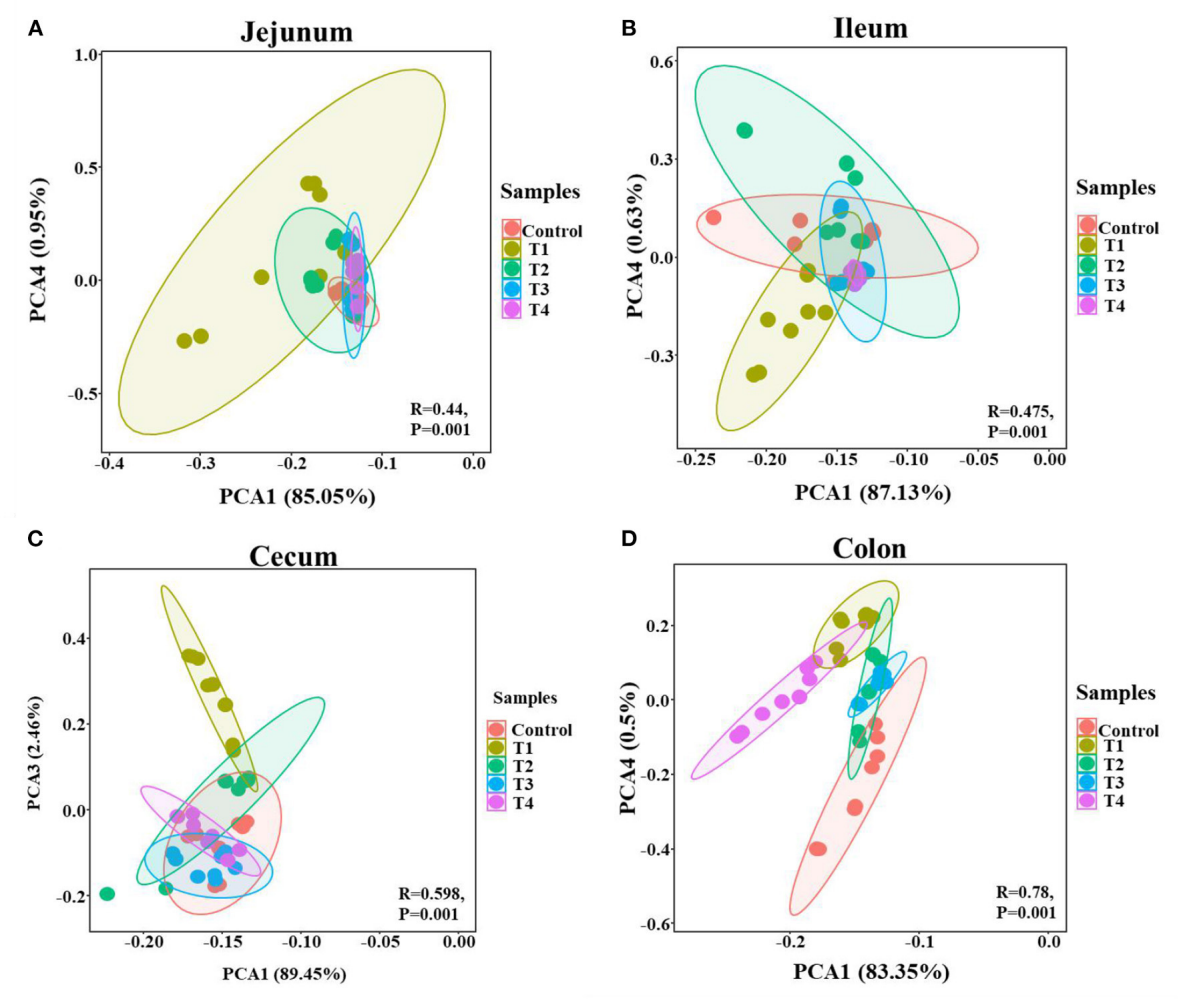
PCA of the mice gut microbial community composition of the Control and T1-4 group based on the Bray-Curtis distances showed distinct clusters, *P*-value and *R*-value were calculated by ANOSIM. The jejunum **(A)**, ileum **(B)**, cecum **(C)**, and colon **(D)** microbial community structure between the Control group and T1–4 groups were differentiated by colors (red, brown, green, blue, purple, respectively).

To validate that sucralose administration will change the structure of gut microbiome in mice, the jejunal, ileal, cecal, colonic contents were collected for 16S rRNA gene sequencing. The alpha-diversity indicated that the number of features and Shannon index had an upward trend from the Control group to T1 and T2 groups, and there was a downward trend from T2 group to T3 and T4 group in mice jejunum (Figures 2A,B), ileum (Figures 2C,D), cecum (Figures 2E,F) and colon (Figures 2G,H). The beta-diversity showed significant changes in mice gut microbiome community membership and structure from Control group to T1-4 groups. Especially in T1 group (Figures 3A–D), its clustering is far away from all other groups in jejunum (Figure 3A) ileum (Figure 3B) cecum (Figure 3C) and colon (Figure 3D). In mice jejunum, ileum and cecum, T3 and T4 groups were closer to control group. In colon, T4 group was as far away from the control group as T1 group.

### The Mice Gut Core Microbiome

**Figure 4:**
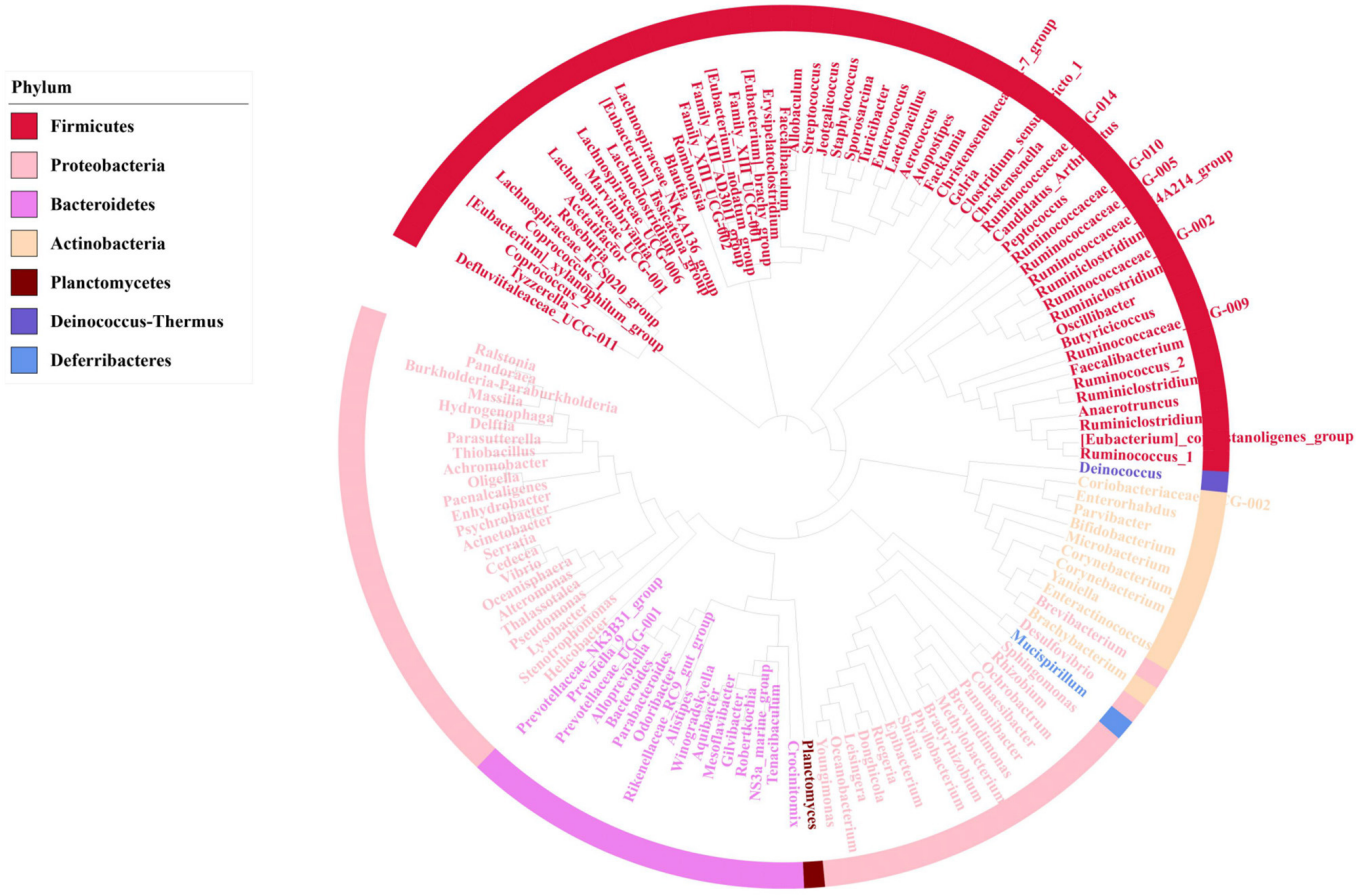
Phylogenetic tree analysis showing top 129 bacterial taxa based on 16S rRNA gene V4-V5 hypervariable regions, after removing 21 features which were uncultured or no rank in genus level. The innermost clades and labels were colored by genus.

To identify the core microbiome in mice gut, Top 51 bacterial features of mice gut core microbiome was obtained by referring the research of Li (23). Most of these features are associated with the phylum Firmicutes (*n* = 26), Bacteroidetes (*n* = 14). At the family level, the top three families were Erysipelotrichaceae (*n* = 10), Lactobacillaceae (*n* = 6) Staphylococcaceae (*n* = 4). The top feature was Allobaculums (*n* = 4) (F2, F20, F49, F66) at genus level. These features sequence and taxonomy are shown in Table 1. Phylogenetic tree analysis indicated the Firmicutes had the highest level of abundance in the phylum, the next was Proteobacteria based on the top 129 genus level (Figure 4).

**Table 1:** The mice core gut microbiome.

**Feature#**	**Feature ID**	**Phylum**	**Order**	**Family**	**Genus**	**Species**
F1	6b16e3df5b1a43f80f1abba36a2f4fa4	Firmicutes	Erysipelotrichales	Erysipelotrichaceae		
F2	c7a8646670d35169426746bafae12863	Firmicutes	Erysipelotrichales	Erysipelotrichaceae	Allobaculums	
F4	e91c5ab4a5ff57293c61ab5f8af8f857	Bacteroidetes	Bacteroidales	S24-7		
F5	2baa2ccaf423b8f4b575c26dd5528527	Firmicutes	Bacillales	Staphylococcaceae	Staphylococcus	
F9	0e01940bde40f2c0199e553a5a89621f	Firmicutes	Erysipelotrichales	Erysipelotrichaceae	Turicibacter	
F10	db763fd81e8bbffe8d937b0b8e34ef3c	Firmicutes	Bacillales	Staphylococcaceae	Staphylococcus	
F3	74fe5a07ff7883bf6065905ae09dab02	Firmicutes	Erysipelotrichales	Erysipelotrichaceae	Faecalibaculum	
F12	daa7e3c372cba75996978c9413cb8023	Bacteroidetes	Bacteroidales	Porphyromonadaceae	Parabacteroides	
F6	bad7a42c2b923635697a99bfd9cfb4d4	Firmicutes	Lactobacillales	Lactobacillaceae	Lactobacillus	
F20	dee4854053933a4ec92f2ab0408b6617	Firmicutes	Erysipelotrichales	Erysipelotrichaceae	Allobaculum	
F16	46f1c0f94998484d634272566ab9045e	Bacteroidetes	Bacteroidales	Rikenellaceae	Alistipes; s	
F34	35af66e08002462940c4550f2caaae05	Proteobacteria	Rhodobacterales	Rhodobacteraceae	Donghicola	
F28	6e1541c94d068be4013d732546963c3b	Bacteroidetes	Bacteroidales	S24-7		
F42	98db5cc259f3b66be220f159b72736e0	Proteobacteria	Burkholderiales	Comamonadaceae	Delftia	
F49	2b380f7d47d59b2b9775fbb9c4d27b2e	Firmicutes	Erysipelotrichales	Erysipelotrichaceae	Allobaculum	
F52	8731e41abac7051ee170aea30cff35cd	Firmicutes	Clostridiales	Lachnospiraceae	Lachnoclostridium	
F45	1e7e2fecb3499fe7acf1ec450f55ae46	Firmicutes	Lactobacillales	Streptococcaceae	Streptococcus	
F50	ed18d5fd0e21931814692926017a6c25	Firmicutes	Clostridiale	Lachnospiraceae	NK4A136	
F39	1ec4262624d166b77c644117324ece51	Bacteroidetes	Bacteroidales	Porphyromonadaceae	Odoribacter	
F13	b27d135cb75eb333fb6d6e29f9496218	Firmicutes	Erysipelotrichales	Erysipelotrichaceae		
F41	bb96c3f96496dd3436c48cc3fe9b869b	Actinobacteria	Corynebacteriales	Corynebacteriaceae	Corynebacterium1	
F66	d99862e3d320187d202bf5427e084262	Firmicutes;	Erysipelotrichales	Erysipelotrichaceae	Allobaculum	
F46	963f23135f931531c59451f0fbb6e12c	Bacteroidetes	Bacteroidales	Prevotellaceae	UCG-001	
F36	88eacccbc95ec6f6264d25d8143274ec	Bacteroidetes	Bacteroidales	Porphyromonadaceae	Odoribacter	
F68	1bbcf22e72576560caa74a36d1034535	Bacteroidetes	Flavobacteriales	Flavobacteriaceae	Mesoflavibacter	
F60	a2c80a0fefad24ad09383620125620ac	Firmicutes	Clostridiales	Clostridiaceae 1	sensu stricto 1	
F11	b415fc5a8da6294f0a2be791c1763b46	Bacteroidetes	Bacteroidales	Bacteroidaceae	Bacteroides	
F77	49fe5c8102e07ba0b1060fe687e1ba41	Bacteroidetes	Flavobacteriales	Flavobacteriaceae	Mesoflavibacter	
F58	86823ff40593228f03d64f36dfcd0c7c	Firmicutes	Clostridiales	Ruminococcaceae	Ruminiclostridium 9	
F89	65e916ef00eacbed2d5068c2d14835b3	Firmicutes	Bacillales	Staphylococcaceae	Jeotgalicoccus	Halotolerans
F84	0a506ad68d12793df4055a2b76ebe412	Proteobacteria	Pseudomonadales	Pseudomonadaceae	Pseudomonas	
F17	3aa637bd332ff9fce9373b81613ec1e3	Proteobacteria	Burkholderiales	Alcaligenaceae	Parasutterella	
F95	4326af51eda901052e87d2bd8df04fee	Bacteroidetes	Flavobacteriales	Flavobacteriaceae	Mesoflavibacter	
F79	23e7dba569ab918cf641dc7c6a19cdca	Proteobacteria	Rhizobiales	Phyllobacteriaceae	Phyllobacterium	
F19	5358db5bc5ebe904ec7caf97db19ca41	Firmicutes	Erysipelotrichales	Erysipelotrichaceae	Faecalibaculum	
F73	18868dd1c73f1dc845edd0783806a273	Actinobacteria	Coriobacteriales	Coriobacteriaceae	Enterorhabdus	
F44	8094bb6c6dd711371552ae749d34bd2e	Bacteria	Bacteroidales	Rikenellaceae	RC9 gut	
F56	2e2b432ddf60afdd484cd4abd0a34fdb	Deferribacteres	Deferribacterales	Deferribacteraceae	Mucispirillum	
F100	a4177cc2db325e8adfae6d8003be7467	Firmicutes	Clostridiales	Lachnospiraceae	NK4A136	
F116	cdcab14f1a38a841458a3b63cdb952a2	Proteobacteria	Rhodobacterales	Rhodobacteraceae	Ruegeria	
F78	1fb77a25c3217ae147accb95cd6e3db9	Firmicutes	Clostridiales	Lachnospiraceae		
F108	d5ca97f9398c389f305fd8e5a89a3d8b	Firmicutes	Clostridiales	Lachnospiraceae	Marvinbryantia	
F123	e3940000c0af54fe0debb7325bb78c42	Bacteroidetes	Flavobacteriales	Flavobacteriaceae	Tenacibaculum	Litoreum
F144	630af886c8a8b7bbef2df85247c40bb7	Firmicutes	Clostridiales	Ruminococcaceae	Ruminiclostridium	
F133	884647b522ef48acb57a64e71987a20f	Proteobacteria	Pseudomonadales	Moraxellaceae	Enhydrobacter	
F148	e4f8ba7abc5dc204a7d9e55e7db910b6	Firmicutes	Bacillales	Planococcaceae	Sporosarcina	
F112	342bdcf4e03a5fa45327aa587fe1b2ce	Firmicutes	Clostridiales	Peptococcaceae		
F149	c1e75978abce59bd25cbfe9ac36067f2	Bacteroidetes;	Flavobacteriales	Flavobacteriaceae	Winogradskyella	
F132	2e9c75913d6338775f03b74a35fc8ece	Firmicutes	Clostridiales	Ruminococcaceae	Ruminiclostridium	
F137	8c7c75dfb1b25dbf2e80105777369689	Proteobacteria	Burkholderiales	Burkholderiaceae	Pandoraea	Oxalativorans
F167	c851e3a644c834c9a924fa361638b492	Firmicutes	Erysipelotrichales	Erysipelotrichaceae		

**Figure 5:**
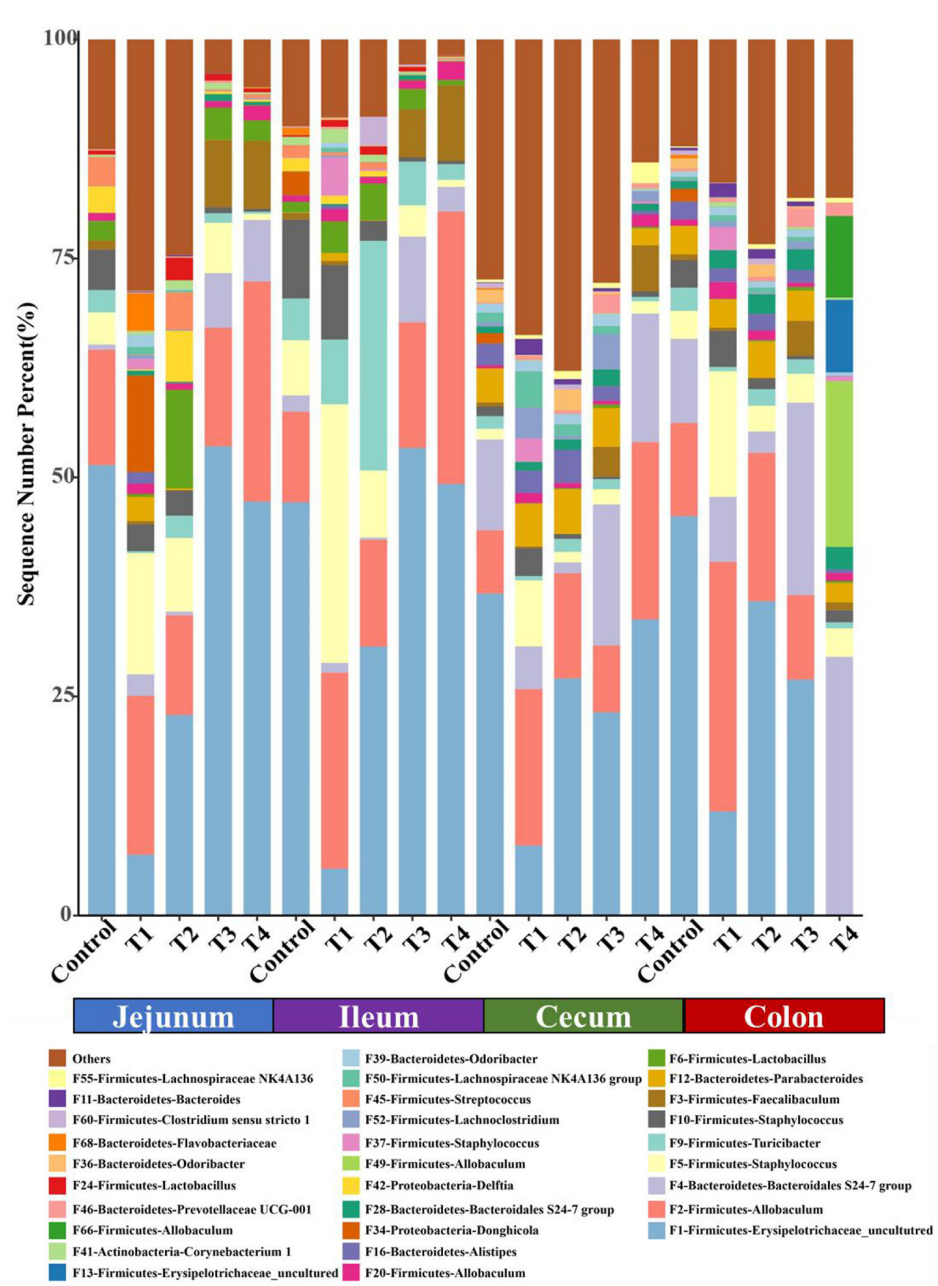
The top 30 features in Control group and T1–4 groups of jejunum, ileum, cecum and colon microbiome in mice. Each color indicates the relative abundance of a bacterial taxon on the bar chart.

We next confirmed the shifts of mice microbiome in different gut segments, top 30 most abundant bacterial features were shown on the bar chart (Figure 5). Among top 5 taxa, in jejunum and cecum, compared with Control group, Firmicutes-Allobaculums (F2) had an upward trend in T1 and T4 group, and it significantly rose in ileum and cecum (*P* < 0.05). In the colon, the T4 group had a significant increase in Firmicutes-Allobaculums (F49 and F66) compared with the Control group. Firmicutes-Staphylococcus (F5) increased significantly in T1 group compared with Control group in four different gut segments (*P* < 0.05) (Figure 5).

### Bacterial Taxa Differentially Represented in Mice Gut Microbiome

**Figure 6:**
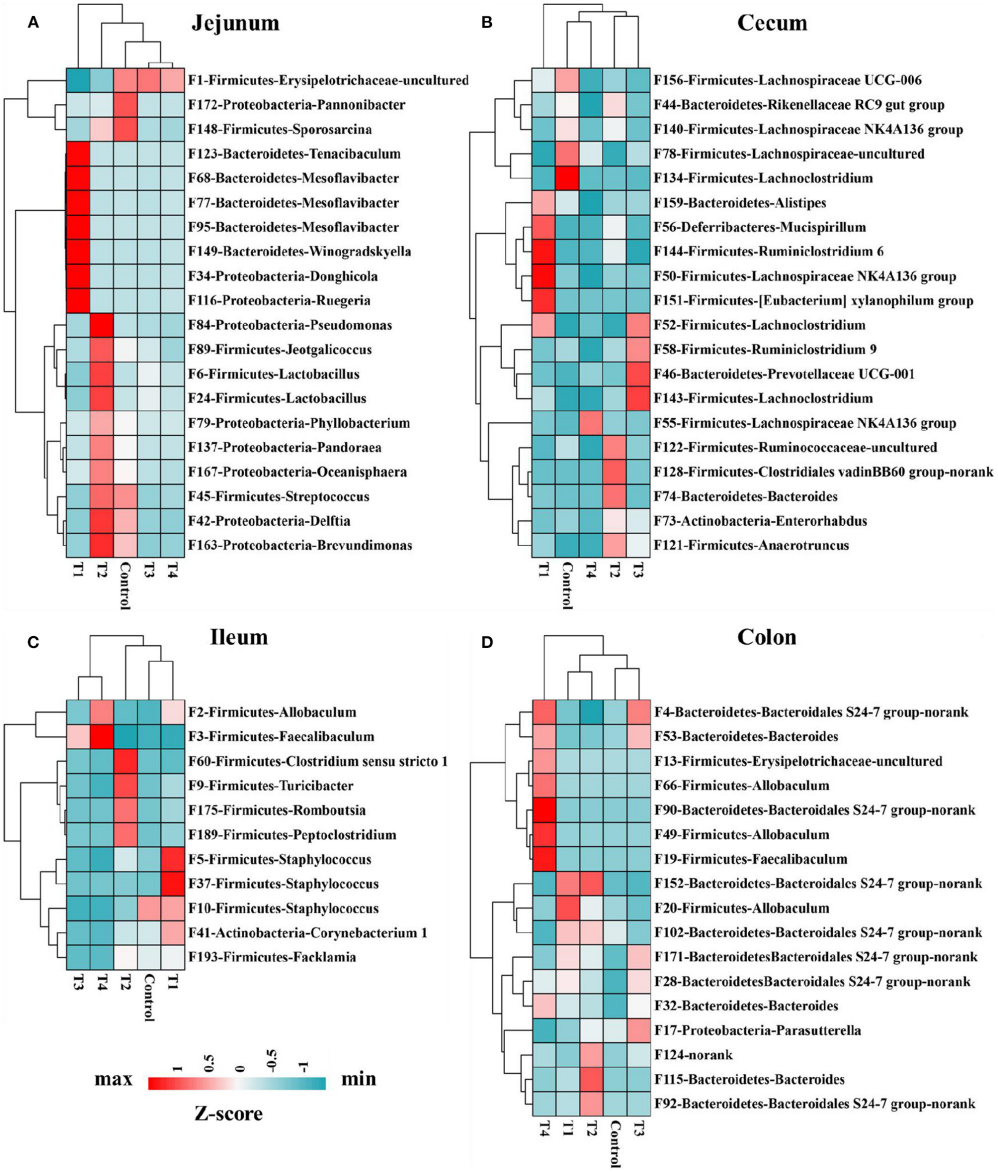
Heat map indicated 68 bacterial taxa were identified by LEfSe (LDA > 3) in mice jejunum (*n* = 20) **(A)**, ileum (*n* = 11) **(C)**, cecum (*n* = 20) **(B)**, and colon (*n* = 17) **(D)** microbiome. The top 1,000 features were used for LEfSe analysis. Heat map shows the average relative abundances on a Z-score.

Mice gut bacterial features were analyzed by using LEfSe (24), the abundance of these significantly different features were shown on the heat map. In jejunum (Figure 6A), Bacteroidetes-Tenacibaculum (F123) and Proteobacteria-Ruegeria (F116) had a significantly increase in T1 group compared with other groups (Control, T2, T3, T4). In ileum (Figure 6C), Firmicutes-Allobaculum (F2) in T1 and T4 group were significantly higher than other group (Control, T2, T3), Firmicutes-Staphylococcus (F5, F37, F10) and Actinobacteria-Corynebacterium 1 (F41) had a significantly increase in T1 group compared with other groups (Control, T2, T3, T4). In cecum (Figure 6B), Firmicutes-Lachnoclostridium (F134) and Firmicutes-Lachnospiraceae UCG-006(F156) in Control group were significantly higher than T1 and T4 group. In colon (Figure 6D), after removing these features which were uncultured or no rank in genus level, we found the Firmicutes-Allobaculum (F20) in T1 group and Firmicutes-Allobaculum (F66, F49) in T4 group increased significantly than other groups (Control, T2, T3).

## Discussion

Sweetness, whether provided by sugar or artificial sweeteners, enhances human appetite and reduce stress, so it is the preference of most people (25, 26). Besides, sucralose is one of the most consumed NNS in the world, since entering the food and beverage market (27), Recent research had shown that sucralose was used instead of sugar to reduce calorie and blood sugar intake (28). However, its effect on human health has been always controversial in recent years. Thus, in the present study, we examined the effects of sucralose (0.0003, 0.003, 0.03, and 0.3 mg/mL) on mice weight. Finally, we found that sucralose administration did not change the phenotype of mice, including the body weight and liver weight. Resent research showing the body weight remained constant by short-term sucralose consumption in human (16). Azad searched Medline, Embase and Cochrane Library for randomized controlled trials that evaluated interventions for NNS, NNS administration had no significant effect on BMI in 1,003 participants (29). This all consistent with our study, and confirms that sucralose as a zero-calorie sweetener does not provide energy to the body.

The intestinal barrier is composed of physical barrier (intestinal epithelium and mucus elements) (30), immunologic (immune cells) (31), and microbial community (32). Intestinal barrier regulates the two-way flow of water, ions and macromolecules between the lumen and the host (33). Epithelial barrier dysfunction has been reported in a variety of intestinal diseases, including inflammatory bowel disease (IBD) and ulcerative colitis (34). With the administration of sucralose, T1-4groups intestinal epithelial barrier was destroyed, as evidenced by the lymphocyte aggregation, especially in T1 and T4 groups. Resent study indicated that high concentrations of sucralose [10 mmol, the public may consume generous sweetener in the diet to achieve up to 10 mmol exposure to sweeteners (35)] induced apoptosis and cell death of intestinal epithelial cells, low concentrations of sucralose (0.1 mmol) down-regulated cell surface claudin 3 (36). Dai et al. (5) research showed that maternal sucralose administration significantly inhibited intestinal development and destroyed the intestinal barrier function in 3-week-old offspring. MUC2 is one of the important products of goblet cells and is closely related to the formation of the mucus layer. Research showed that compared with the control group, the production of MUC2 was significantly decreased in the sucralose group (5). After 6 months of sucralose administration, the genes related to LPS synthesis increased significantly (37). The relative mRNA expression levels of proinflammatory factors, including IL-1β, IFN-γ, and TNF-α were significantly higher in sucralose group than those in control group in colon (5). In our study, sucralose administration induced lymphocyte aggregation, which may lead to the increase of inflammatory factors. These revealed that sucralose administration might disrupt intestinal barrier function, the sucralose concentration and the effect on intestinal barrier of these studies all consistent with T4 group in our study, more interestingly, we also found the intestinal barrier was significantly damaged in group T1.

Gut microbiome is also an important part of the intestinal barrier (38), it is a complex and dynamic system, intestinal imbalanced states or even unhealthy stable states will develop, or potentially lead to diseases, including IBD, Nonalcoholic fatty liver disease (NALFD) and Irritable Bowel Syndrome (IBS) (39, 40). In this study, we demonstrate low dose of sucralose alter gut microbiome in mice by using 16S rRNA gene sequencing, T1-4 groups mice accessed 0.0003 g/mL, 0.003 mg/mL, 0.03 mg/mL, 0.3 mg/mL sucralose for 16 weeks, 0.1 mg/ml of sucralose solution was FDA acceptable daily intake (18). The results showed the number of features and Shannon index had an upward trend in T1 group and a downward trend in T4 group compared with Control group, Beta-diversity indicated T1 group was distinct from other groups, especially Control group. Sánchez-Tapia research found 1.5% (1.5 mg/mL) concentration of sucralose led to the lowest α-diversity in rats gut microbiota, its PCoA analysis revealed that gut microbiota was differentially shifted by sucralose (41). Many previous studies had shown that ADI (0.1 mg/ml) of sucralose significantly altered mice gut microbiome (37, 42). These results were consistent with T4 group in our study, however, the new finding in our research was T1 (0.0003 mg/mL) group, like T4 (0.3 mg/mL), also altered mice gut microbiome.

Core microbiome is essential to understand its function in the gut, it has been well- researched in different species (43, 44). Generally, a core microbiome indicates common bacterial present in all or most (e.g., >90%) of the communities in the host (45). In this study, a total of 51 core microbiome members of mice were identified in five groups. Most of these features are associated with the phylum Firmicutes (*n* = 26), Bacteroidetes (*n* = 14). Research have shown 2.5% sucralose treatment group increased the Firmicutes in phylum level (46). In our study, we found the top feature was Allobaculums (*n* = 4) (F2, F20, F49, F66) at genus level. Besides, Allobaculums of T1 and T4 group were significantly higher in mice jejunum, ileum and colon. Many research had shown Allobaculums significantly increased in diabetes model group compared with normal group (47, 48). However, whether sucralose administration will induce diabetes by altering gut microbiota, it requires further research.

Sucralose intake associated bacterial features were identified by using LEfSe, an algorithm that not only analyze statistical significance but also biological consistency. The results showing, in jejunum, Bacteroidetes-Tenacibaculum (F123) and Proteobacteria-Ruegeria (F116) significantly increased in T1 group compared with Control group. Tenacibaculum is a genus of gram negative, filamentous bacteria, related to the disease (tenacibaculosis) existing in aquaculture farms all over the world (49). Rubio-Portillo research identified Ruegeria OUT was associated with tissue necrosis in their hosts (50). In ileum, Firmicutes-Staphylococcus (F5, F37, F10) and Actinobacteria-Corynebacterium 1 (F41) significantly increased in T1 group compared with Control groups. The representative species of Staphylococcus are Staphylococcus aureus, it is a pathogen that usually colonizes the human anterior nostrils. This pathogen is one of main causes of life-threatening bloodstream infections, as sepsis and endocarditis (51). In cecum, Firmicutes-Lachnoclostridium (F134) and Firmicutes-Lachnospiraceae UCG-006 (F156) significantly decreased in T4 group than Control group. Lachnoclostridium was significantly up-regulated after treatment of obesity and inflammatory bowel disease (IBD) (52, 53). Lachnospiraceae is the major producers of short-chain fatty acids (SCFA), and is significantly related with enhanced gut barrier function (54, 55). In colon, Firmicutes-Allobaculum (F20) in T1 group and Firmicutes-Allobaculum (F66, F49) in T4 group increased significantly than other groups (Control, T2, T3). Allobaculum is not only positive related to diabetes (47, 48), but also related to ileal RORγT and IL-17 levels (56), induced susceptibility to autoimmune encephalitis (57), increased the expansion of inflammatory T helper 17 cells in gut (58). So, low dose of sucralose (T1, 0.0003 g/mL) consumption significantly altered mice gut microbiome, it might contribute to the increased expression of pro-inflammatory, these changes same as most previous study had indicated sucralose intake at human ADI (T4, 0.03 mg/mL) altered the gut microbiome in mice (5, 18, 41, 46).

## Conclusion

Overall, our study demonstrated sucralose administration did not change mice body weight, but low dose of sucralose (0.0003 mg/mL) significantly altered mice gut microbiome, including the increases of Tenacibaculum, Ruegeria, Staphylococcus and Allobaculum in genus level in mice jejunum, ileum and colon. The decrease of Lachnoclostridium and Lachnospiraceae in cecum of T4 group mice. Although the sucralose of ADI (0.3 mg/mL) level also altered the gut microbiome in mice, the human daily intake of sucralose is usually lower than this concentration. We should focus on the low dose of sucralose administration in human. Finally, our research is limited to the effect of low-dose sucralose on the gut microbiome of mice, and the relevance to human metabolic diseases warrant further investigation.

## Data Availability Statement

The datasets presented in this study can be found in online repositories. The names of the repository/repositories and accession number(s) can be found below: https://www.ncbi.nlm.nih.gov/, PRJNA787401.

## Ethics Statement

The animal study was reviewed and approved by the Institutional Animal Care and Use Committee of Zhejiang Academy of Agricultural Sciences.

## Author Contributions

ZZ, YX, YR, and JL designed the experiment. ZZ, LM, HP, and XW conducted the animal experiments. ZZ, YX, LM, YR, and JL wrote and revised the manuscript. ZZ, YX, LM, YR, and JL did experimental analysis, collected, and analyzed the data. All authors reviewed the manuscript and contributed to the article and approved the submitted version.

## Funding

This work was financially supported by the State Key Laboratory for Managing Biotic and Chemical Threats to the Quality and Safety of Agroproducts, Grant/Award Number: 2010DS700124-ZZ2017, the Open Project of Hubei Key Laboratory of Animal Nutrition and Feed Science (No. 201806), and the National Natural Science Foundation of China (31972999).

## Conflict of Interest

The authors declare that the research was conducted in the absence of any commercial or financial relationships that could be construed as a potential conflict of interest.

## Publisher's Note

All claims expressed in this article are solely those of the authors and do not necessarily represent those of their affiliated organizations, or those of the publisher, the editors and the reviewers. Any product that may be evaluated in this article, or claim that may be made by its manufacturer, is not guaranteed or endorsed by the publisher.
